# MFR‐UNet: A Medical Image Segmentation Network With Fused Multi‐Scale Feature Refinement

**DOI:** 10.1049/syb2.70049

**Published:** 2025-12-24

**Authors:** Shaoqiang Wang, Guiling Shi, Shuo Sun, Yuchen Wang, Yulin Zhang, Weixian Li, Yawu Zhao, Xiaochun Cheng

**Affiliations:** ^1^ Qingdao University of Technology Qingdao Shandong China; ^2^ Shandong University of Science and Technology Qingdao Shandong China; ^3^ Langfang Normal University Langfang Hebei China; ^4^ School of Medical Informational Engineering Shandong University of Traditional Chinese Medicine Jinan Shandong China; ^5^ Computer Science Department Bay Campus Swansea University Swansea Wales UK

**Keywords:** feature fusion, large receptive field, medical image segmentation, wavelet

## Abstract

Medical image segmentation is crucial for clinical diagnosis and treatment planning. Although methods based on CNN, particularly U‐Net and its variants, have achieved remarkable success in automated segmentation tasks, they still face challenges in effectively capturing long‐range dependencies, refining multi‐level features, and efficiently integrating cross‐level information. To address these issues, we propose a novel U‐Net architecture incorporating a multi‐scale feature refinement mechanism (MFR‐UNet). This network enhances segmentation accuracy and robustness by integrating three innovative modules. First, we designed a wavelet transform convolution (WtConv) module. By decomposing, processing, and reconstructing features in the frequency domain, this module enables the model to learn high‐frequency details and low‐frequency contours with greater precision. Second, we introduce a large receptive field attention (LRFA) module in the encoder. Combining deep separable convolutions with multi‐head attention, LRFA efficiently captures global contextual information at low computational cost. Finally, in the skip connections and decoding path, our weighted contextual fusion module (WCF) module dynamically generates channel attention weights for one feature stream to another, achieving efficient adaptive feature fusion. Simulation experiments on multiple public medical image segmentation datasets demonstrate that our MFR‐UNet outperforms several existing mainstream methods in key metrics such as Dice coefficient and IoU, proving its effectiveness in enhancing segmentation accuracy and boundary clarity.

## Graphical abstract

Addressing the challenges of CNN‐based methods (especially U‐Net and its variants) in medical image segmentation—such as difficulties in capturing long‐range dependencies and insufficient refinement of multi‐scale features—this paper proposes the MFR‐UNet architecture integrated with a multi‐scale feature refinement mechanism, which enhances segmentation accuracy and robustness via three innovative modules. Experiments on multiple public datasets demonstrate that MFR‐UNet outperforms existing mainstream methods in key metrics such as Dice coefficient and IoU, effectively improving the accuracy and boundary clarity of medical image segmentation.

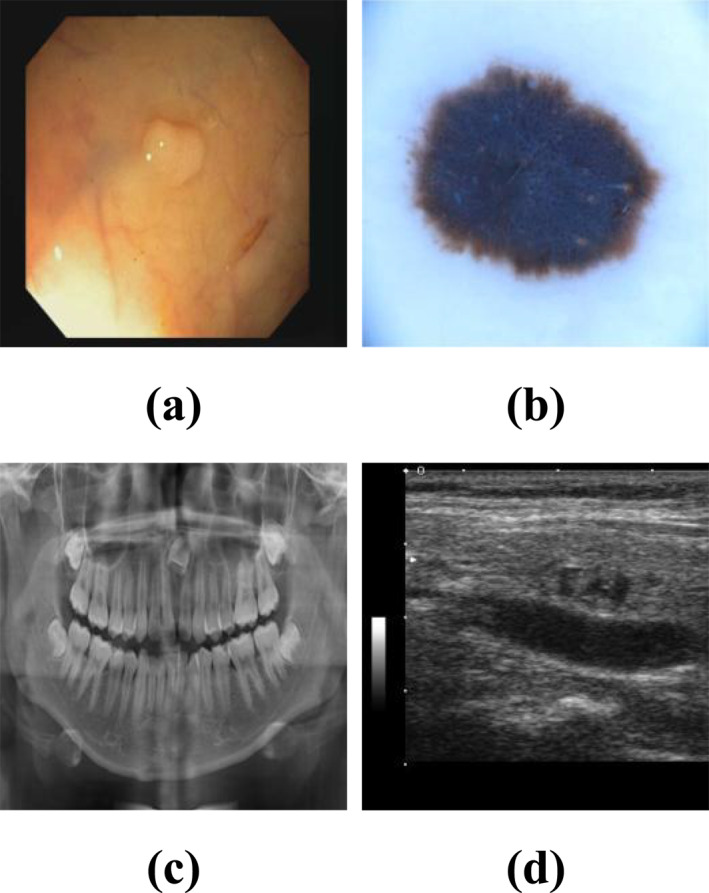

## 1 Introduction

**FIGURE 1:**
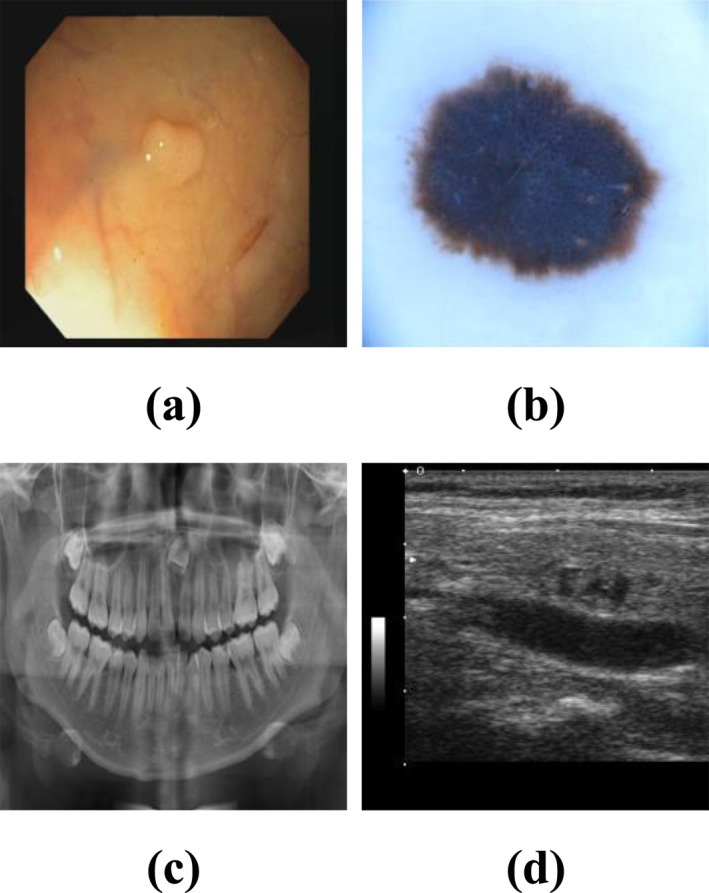
(a) A colonoscopy polyp image from the CVC‐ClinicDB dataset; (b) A dermoscopy lesion image from the ISIC 2017 dataset; (c) A dental x‐ray image from the MICCAI Tooth dataset; (d) A thyroid nodule ultrasound image from the DDTI dataset.

Precise tumour segmentation is a critical step in surgical planning and radiation therapy [1]. Its accuracy directly impacts treatment strategy formulation and patient prognosis. Medical image segmentation, serving as the pivotal technology bridging medical imaging and clinical decision‐making, aims to automatically identify and delineate anatomical structures or pathological regions within images through computational algorithms [2, 3]. From early traditional methods based on thresholding or region growing to today's pixel‐level intelligent analysis powered by deep learning, this technology has significantly enhanced the efficiency, precision and reproducibility of image analysis, becoming an indispensable component of modern precision medicine. However, designing segmentation models with robust generalisation capabilities remains challenging due to differences in imaging principles across modalities and the vast variability in lesion location, morphology, size and boundary clarity [4, 5], as shown in Figure 1.

Among numerous deep learning models, CNN based on U‐shaped architectures have achieved landmark success in medical image segmentation through their symmetric encoder–decoder structure and unique skip‐connection mechanism [6]. However, CNN rely on local convolutional kernels to process information, with their receptive fields expanding far slower than the increasing network depth [7, 8]. This inherent local bias makes it difficult for models to capture correlations between anatomical structures spanning large spatial distances within images. This represents a significant limitation for segmentation tasks requiring global context understanding, such as segmenting irregularly shaped or widely distributed lesions. Moreover, U‐Net's skip connections typically employ simple feature concatenation, directly combining high‐resolution detail features from shallow layers with low‐resolution semantic features from deep layers. Although effective, this approach lacks adaptability, potentially leading to semantic conflicts or feature redundancy, and cannot guarantee optimal fusion of features from different hierarchical levels [9].

Subsequent studies, such as attention U‐Net, which enhances responses to critical regions by introducing attention gates, or hybrid models like TransUNet that incorporate transformers to capture global information, have partially mitigated these issues [10, 11]. However, they have not fundamentally resolved the core contradiction between deep information degradation and inefficient global modelling. For instance, simple attention mechanisms remain built upon local features, whereas standard transformers face quadratic computational complexity and may disrupt spatial structural continuity at extremely low feature map resolutions because of their lack of spatial inductive bias.

To address these core challenges, we propose a novel U‐Net architecture, MFR‐UNet, which integrates a multi‐scale feature refinement mechanism. This network fundamentally enhances the expressive power of deep features by incorporating three innovative synergistic modules at key positions within the U‐Net architecture, thereby overcoming the performance bottlenecks of existing models. Our main contributions are as follows:

- **Wavelet transform convolution module.** This module is integrated into the deep layers of the encoder. It decomposes feature maps into the frequency domain via discrete wavelet transform, enabling separate processing of high‐frequency details and low‐frequency contour information. This approach effectively enhances the model's ability to preserve edges and fine textures.
- **Large receptive field attention module.** This module efficiently expands the receptive field through parallel separable convolutions and explicitly models long‐range dependencies using a multi‐head attention mechanism. This design effectively addresses the insufficient global context awareness of traditional CNNs while maintaining low computational cost.
- **Weighted contextual fusion module.** This module is employed to optimise the feature fusion process in skip connections. It dynamically generates channel attention weights for one decoder feature stream from another encoder feature stream, enabling adaptive and efficient fusion of cross‐level features while effectively suppressing feature redundancy and semantic conflicts.

## 2 Related Work

### 2.1 CNN for Image Segmentation

Convolutional neural networks have become the cornerstone of medical image segmentation because of their powerful hierarchical feature extraction capabilities. Among these, the U‐Net architecture proposed by Ronneberger et al. [12, 13] stands out. Through its symmetric encoder‐decoder structure and innovative skip connections, it effectively integrates low‐level details with high‐level semantics, setting new benchmarks for high‐precision segmentation tasks.

Building upon U‐Net, researchers have pursued expansions and optimisations across multiple dimensions. U‐Net++ introduces nested and dense skip connections, constructing multi‐level feature aggregation paths within the decoder to enhance information flow between feature maps at different scales [14]. This approach demonstrates higher accuracy when processing organs with complex morphologies, such as kidneys and livers. ResU‐Net integrates residual learning units into both the encoder and decoder of U‐Net [15], effectively mitigating gradient vanishing issues that may arise with increasing network depth, enabling training of deeper architectures. To enable adaptive focus on critical regions, Attention U‐Net introduces an attention gate mechanism positioned on skip‐connection paths [16]. This gate automatically learns and amplifies task‐relevant feature regions based on high‐level semantic information while suppressing background noise and irrelevant tissue interference. These enhancements significantly boost CNN performance for specific tasks.

However, all CNN‐based architectures share an inherent limitation: the locality of convolution kernels. Standard convolution operations can only process information within the receptive field, making it difficult for models to capture long‐range dependencies between anatomical structures. Although expanding the receptive field through stacking more convolution layers or using dilated convolution can be attempted, these approaches often lead to a steep increase in computational cost or result in the gridding effect in feature maps, failing to fundamentally resolve the issue.

### 2.2 Transformer for Image Segmentation

To overcome the locality constraints of CNN, the research community turned its attention to the transformer architecture, which had initially achieved tremendous success in the NLP domain [17]. Its core self‐attention mechanism computes pairwise relationships between all elements in the input sequence, thereby capturing global dependencies. Vision transformer represents pioneering work applying transformers to image recognition [18, 19]. It segments images into a sequence of fixed‐size patches, linearly embeds these patches, and feeds them as a sequence into the transformer encoder.

TransUNet pioneered the integration of ViT with the U‐Net architecture for medical image segmentation [20]. It leverages CNN to extract shallow features, serialises the feature maps, feeds them into the transformer encoder to model global context and finally restores spatial resolution through a decoder and skip connections. This design demonstrated the transformer's immense potential for capturing long‐range anatomical correlations. Subsequently, Swin‐Unet introduced windowed self‐attention and shifted window mechanisms from Swin transformers [21, 22], confining self‐attention computations to non‐overlapping local windows while enabling cross‐window information exchange. This hierarchical design significantly reduces computational complexity, enabling high‐resolution image processing while preserving robust global modelling capabilities [23].

Despite Transformers' excellence in modelling global dependencies, they face limitations. First, the computational complexity of its global self‐attention scales quadratically with the input sequence length, making it computationally expensive for high‐resolution medical images. Second, Transformers lack the inductive biases inherent in CNN, such as locality and translation invariance. This necessitates extensive pre‐training on large‐scale datasets to achieve optimal performance, yet high‐quality large‐scale annotated medical datasets are often difficult to obtain.

### 2.3 CNN and Transformer for Image Segmentation

To balance the local feature extraction efficiency of CNN with the global context modelling capability of transformers, hybrid architectures emerged and quickly became a research hotspot. These models typically follow a “local‐global” collaborative processing design philosophy, aiming to achieve complementary advantages between the two architectures [24].

Typical hybrid models employ CNN in the shallow layers of the encoder to efficiently extract low‐level details such as texture and edges from images. Transformer modules are then introduced in the deeper layers to capture long‐range structural relationships between organs or between pathological regions and surrounding tissues. For instance, the TransFuse model employs a dual‐branch architecture that processes feature streams from CNN and transformers in parallel, facilitating information exchange across multiple levels through a specially designed BiFusion module. MedT proposes a gated axial‐attention model that retains the CNN backbone structure while selectively introducing global context by computing attention across different axes, thereby balancing local and global information [25].

Additionally, Qiao et al. designed the multi‐scale gated axial transformer (MSGATNet) [26]. This network innovatively combines axial Transformers with multi‐scale gating mechanisms: the former captures image features along both horizontal and vertical dimensions, while the latter dynamically adjusts information flow between different scales. This design elegantly balances the preservation of structural details with the modelling of cross‐scale semantics. Recently, Zhao et al. proposed the three‐path feature incremental attention network (TPFIANet) [27]. By constructing a parallel multi‐branch fusion architecture that alternately embeds convolutional and attention modules, it efficiently captures features across different levels and scales, further enhancing accuracy, robustness, and efficiency in medical image segmentation tasks. These innovations continuously expand the application boundaries of CNN‐Transformer fusion architectures, propelling them to new heights in model complexity, task generalisation and real‐world applicability.

Despite the remarkable success of hybrid architectures across numerous tasks, designing optimal fusion strategies remains an open challenge. Simple feature concatenation or addition may fail to effectively align and fuse features from two heterogeneous models, potentially introducing noise. Furthermore, although complex fusion modules often yield superior performance, they typically increase model parameters and computational complexity. Therefore, designing a compact, efficient fusion architecture that requires no additional supervision represents a crucial future research direction. The MFR‐UNet proposed in this paper advances this goal by systematically optimising the U‐Net architecture through a series of ingeniously designed functional modules.

## 3 Our Proposed MFR‐UNet

### 3.1 Wavelet Transform Convolution Module

**FIGURE 2:**
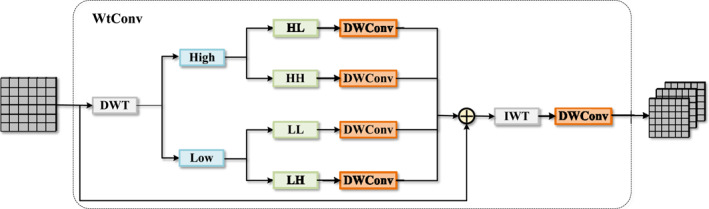
Details of our proposed wavelet transform convolution module.

To perform a more refined analysis of features in the frequency domain, we introduce the wavelet transform convolution (WtConv) module [28]. As shown in Figure 2, the core idea of this module is to utilise the discrete wavelet transform (DWT) to decompose the feature map into different frequency components, process these components independently, and then reconstruct them using the inverse wavelet transform (IWT). This design enables the model to separately attend to and learn high‐frequency details and low‐frequency contour information.

Given an input feature map $X\in R^{H\times W\times C}$, the WtConv module first applies the DWT using the Haar wavelet basis to decompose it into four sub‐bands:

(1) $$X\overset{\text{DWT}}{\to }\left\{X_{LL},X_{LH},X_{HL},X_{HH}\right\}$$

Here, $X_{LL}$ represents the low‐frequency component, capturing the approximate or contour information of the feature map. $X_{LH}$, $X_{HL}$, and $X_{HH}$ represent the high‐frequency components in the horizontal, vertical and diagonal directions, respectively, containing the detail and edge information of the feature map.

After decomposition, each sub‐band is fed into an independent depth‐wise convolution (DWConv) layer for processing. This frequency‐separated processing allows the model to learn specific patterns for different frequency components; for example, one branch might focus on texture details, whereas another concentrates on overall structure. This process can be represented as follows:

(2) $$Y_{\text{sub}}={\text{DWConv}}_{\text{sub}}\left(X_{\text{sub}}\right),\;\text{for}\;\text{sub}\in \left\{LL,LH,HL,HH\right\}$$

Subsequently, all processed sub‐band features are aggregated through element‐wise summation and then reconstructed back to the spatial domain using IWT:

(3) $$Y_{\text{rec}}=\text{IWT}\left(\sum_{\text{sub}}Y_{\text{sub}}\right)$$

Finally, to further integrate the reconstructed features, we apply an additional DWConv layer to produce the module's final output Y. This step helps to smooth out artefacts that may be introduced by the wavelet reconstruction and promotes the fusion of information from different frequencies.

(4) $$Y={\text{DWConv}}_{\text{final}}\left(Y_{\text{rec}}\right)$$

In this way, the WtConv module can perform a deep analysis and processing of features within the frequency domain in a computationally efficient manner, thereby enhancing the model's ability to understand complex scenes.

### 3.2 Large Receptive Field Attention Module

**FIGURE 3:**
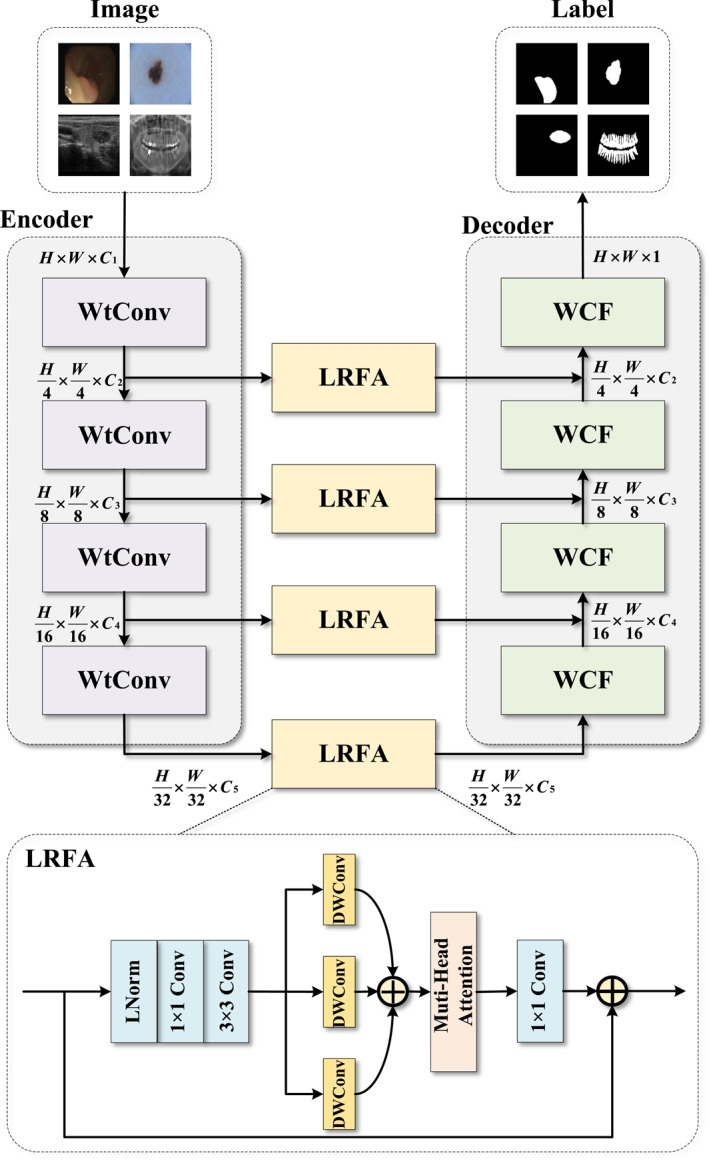
The overall framework of the medical image segmentation model MFR‐UNet.

To effectively capture multi‐scale contextual information and long‐range dependencies in images, we have designed a novel hybrid computational unit named the large receptive field attention (LRFA) module. The overall architecture of the LRFA module is illustrated in Figure 3. Its core idea is to combine the local feature extraction capability of convolutions with the global modelling ability of the self‐attention mechanism.

As shown in Figure 3, the input feature map X is first passed through a pre‐processing unit, which consists of a layer normalisation (LN) layer followed by a 1×1 and a 3×3 convolutional layer in series to extract robust local representations. This process can be formalised as follows:

(5) $$X_{\text{local}}={\text{Conv}}_{3\times 3}\left(\sigma \left({\text{Conv}}_{1\times 1}\left(\text{LN}\left(X\right)\right)\right)\right)$$

where σ represents the GELU non‐linear activation function.

Next, to expand the receptive field without significantly increasing computational cost, we feed the extracted local features $X_{\text{local}}$ into three parallel depth‐wise convolution (DWConv) branches for multi‐scale receptive field aggregation. The aggregated feature $X_{\text{agg}}$ is obtained by element‐wise summation of the outputs from each branch:

(6) $$X_{\text{agg}}=\sum_{i=1}^{3}{\text{DWConv}}_{i}\left(X_{\text{local}}\right)$$

Subsequently, the aggregated feature $X_{\text{agg}}$ is fed into a standard multi‐head self‐attention (MHA) module for feature refinement. This step aims to explicitly model the pairwise relationships between all spatial positions in the feature map, enabling the model to dynamically and non‐locally enhance more informative feature regions. Its output is denoted as $X_{\text{attn}}=\text{MHA}\left(X_{\text{agg}}\right)$.

Finally, the output of the module is completed through a main residual connection. The attention‐refined feature $X_{\text{attn}}$ is first passed through a 1×1 convolution for channel‐wise information integration, and the result is then added to the original module input X to obtain the final output Y:

(7) $$Y=X+{\text{Conv}}_{1\times 1}\left(X_{\text{attn}}\right)$$

This residual structure ensures effective information flow and stable gradient backpropagation, allowing the LRFA module to be easily integrated into any deep neural network architecture.

### 3.3 Weighted Contextual Fusion Module

To achieve effective information interaction and fusion between different feature streams, we propose a weighted contextual fusion (WCF) module. This module aims to dynamically generate channel attention weights for one feature stream (e.g., features from the decoder) by utilising another feature stream (e.g., features from the encoder), thereby fusing complementary information in an adaptive manner. The WCF module receives two feature maps as input, which we denote as $X_{1}$ and $X_{2}$, respectively.

**FIGURE 4:**
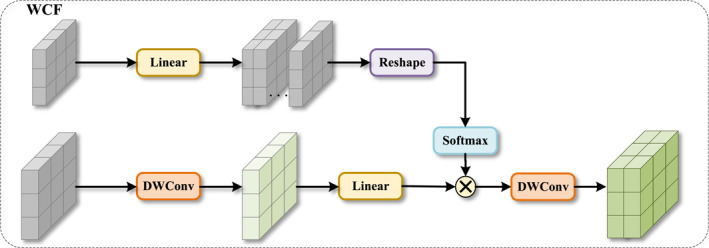
Details of our proposed weighted contrastive fusion module.

As shown in Figure 4, the computation process of the module is mainly divided into two parallel branches. In the dynamic weight generation branch, the input feature $X_{1}$ is used to generate channel attention weights. It is first transformed by a linear layer, then adjusted to a suitable dimension through a reshape operation, and finally, the Softmax function is applied to compute the normalised attention weights $W_{\text{attn}}$.

(8) $$W_{\text{attn}}=\text{Softmax}\left(\text{Reshape}\left(\text{Linear}\left(X_{1}\right)\right)\right)$$

In the parallel feature transformation branch, the other input feature $X_{2}$ is first passed through a depth‐wise convolution (DWConv) layer to extract spatial features, and then transformed in the channel dimension by a linear layer to obtain the features to be weighted, $F_{\text{in}}$.

(9) $$F_{\text{in}}=\text{Linear}\left(\text{DWConv}\left(X_{2}\right)\right)$$

In the fusion stage, we perform element‐wise multiplication between the dynamically generated attention weights $W_{\text{attn}}$ and the features to be weighted, $F_{\text{in}}$. This operation can be understood as using the information from $X_{1}$ to dynamically and selectively enhance or suppress different feature channels of $X_{2}$. Finally, the fused feature $F_{\text{fused}}$ is passed through another DWConv layer for final feature integration, producing the module's output Y.

(10) $$F_{\text{fused}}=W_{\text{attn}}⊗F_{\text{in}}$$

(11) $$Y=\text{DWConv}\left(F_{\text{fused}}\right)$$

Through this weighted fusion mechanism, the WCF module can flexibly integrate features from different sources and highlight the most important information for the current task.

## 4 Experimental Results Analysis

### 4.1 Datasets

To comprehensively evaluate the performance and generalisation capabilities of our proposed MFR‐UNet, we conducted experiments using four publicly available datasets spanning different imaging modalities. These datasets enable thorough testing of the model's performance across diverse scenarios.

#### 4.1.1 CVC‐ClinicDB

This dataset comprises 612 frames extracted from colonoscopy videos, each annotated with a polyp region segmentation mask by professional physicians. It serves as a common benchmark for evaluating endoscopic polyp segmentation algorithms (Download link: https://paperswithcode.com/dataset/cvc‐clinicdb).

#### 4.1.2 ISIC 2017

This large‐scale dermatoscopy dataset comprises 2000 training images designed to support research on identifying and segmenting skin lesions such as melanoma. Each image provides pixel‐level segmentation annotations for lesion areas, presenting challenges due to the diverse lesion morphologies and frequently indistinct boundaries with surrounding skin (Download link: https://challenge.isic‐archive.com/data/).

#### 4.1.3 DDTI (Diagnostic Dataset for Thyroid Imaging)

This dataset focuses on thyroid ultrasound images, featuring various types of thyroid nodules (benign and malignant) alongside normal thyroid tissue images. Inherent challenges in ultrasound imaging—low contrast, speckle noise and blurred nodule boundaries—pose significant difficulties for precise segmentation, making it a crucial dataset for testing model robustness (Download link: https://www.kaggle.com/datasets/dasmehdixtr/ddti‐thyroid‐ultrasound‐images).

#### 4.1.4 MICCAI Tooth

This dataset originates from the MICCAI 2D tooth segmentation challenge, providing a large collection of dental x‐ray images with corresponding segmentation masks. It aims to evaluate model performance on segmenting high‐density, finely structured and densely packed tissues such as teeth (Download link: https://tianchi.aliyun.com/dataset/156596).

### 4.2 Evaluation Metrics

To comprehensively and objectively evaluate the performance of the proposed model, this study selects four widely used evaluation metrics in this field. They are all calculated based on True Positives (TP), False Positives (FP), and False Negatives (FN).

- **Dice Similarity Coefficient (DSC):** This is the most commonly used metric for measuring the overlap between the predicted segmentation region and the ground truth region, and it is particularly robust for targets of varying sizes. Its value ranges from [0, 1], with a value closer to 1 indicating a better segmentation result.

(12) $$\text{DSC}=\frac{2\times \text{TP}}{2\times \text{TP}+\text{FP}+\text{FN}}$$

- **Intersection over Union (IoU):** Also known as the Jaccard index, it evaluates segmentation performance by calculating the ratio of the intersection to the union of the predicted and ground truth regions. It is more sensitive to the accuracy of segmentation boundaries than the Dice coefficient.

(13) $$\text{IoU}=\frac{\text{TP}}{\text{TP}+\text{FP}+\text{FN}}$$

- **Precision:** This metric measures the proportion of pixels that are correctly predicted as the target region among all pixels predicted as the target region by the model. High precision implies a low false positive rate, which is crucial for avoiding unnecessary clinical interventions.

(14) $$\text{Precision}=\frac{\text{TP}}{\text{TP}+\text{FP}}$$

- **Sensitivity (or Recall):** This metric measures the proportion of pixels that are successfully predicted by the model among all pixels in the actual target region. High sensitivity implies a low false negative rate, which is essential to ensure that no small lesions are missed.

(15) $$\text{Sensitivity}=\frac{\text{TP}}{\text{TP}+\text{FN}}$$

### 4.3 Implementation Details

#### 4.3.1 Experimental Environment

All experiments were conducted in a unified software and hardware environment to ensure the reproducibility of the results. Our hardware platform was a server equipped with an NVIDIA RTX 4090 GPU (24 GB VRAM), running the Ubuntu 20.04 operating system. The deep learning framework used was PyTorch 2.1, with CUDA 12.3 for GPU acceleration.

#### 4.3.2 Data Preprocessing and Augmentation

Before being fed into the network, all input images and their corresponding masks were uniformly resized to 256×256 pixels. We normalised the images by subtracting the mean and dividing by the standard deviation to accelerate model convergence. To enhance the model's generalisation ability and mitigate overfitting, we employed a series of online data augmentation strategies, including: random rotation (from −15 to +15°), random horizontal and vertical flips, random scaling (from 0.8 to 1.2 times) and elastic transformations.

#### 4.3.3 Training Configuration

We utilised the AdamW optimiser for updating the model parameters, with an initial learning rate set to $1\times 10^{-4}$ and a weight decay of $1\times 10^{-5}$. The learning rate was dynamically adjusted during training using a cosine annealing schedule, which smoothly decreases the learning rate over time. The model was trained for a total of 200 epochs, with a batch size of 8. To ensure a robust and reliable performance evaluation, we employed a five‐fold cross‐validation scheme to make full use of the dataset.

#### 4.3.4 Loss Function

To effectively address the potential class imbalance problem in medical image segmentation and to enhance the overall performance of the model, this study employs a hybrid loss function that combines the cross‐entropy Loss $\left(L_{CE}\right)$ and the Dice Loss $\left(L_{\mathrm{Dice}}\right)$. The total loss, $L_{\text{total}}$, is defined as follows:

(16) $$L_{\text{total}}=\lambda_{1}L_{CE}+\lambda_{2}L_{\mathrm{Dice}}$$

where $L_{CE}$ focuses on pixel‐level classification accuracy, whereas $L_{\mathrm{Dice}}$ directly optimises the overlap between the prediction and the ground truth. In this study, we empirically set the weighting coefficients to $\lambda_{1}=0.4$ and $\lambda_{2}=0.6$. This choice appropriately increases the weight of the Dice loss to more directly optimise for structural similarity in the segmentation, which is particularly beneficial for improving the model's ability to learn small targets and fine‐grained boundary details.

### 4.4 Results and Discussion

To systematically validate the effectiveness of our proposed MFR‐UNet, we conducted extensive comparisons against multiple state‐of‐the‐art segmentation methods, including the classic U‐Net, U‐Net++, AttUnet, UNeXt, WRANet, DualA‐Net, DPMNet and TPFIANet. All models were trained and evaluated under identical experimental settings across four distinct multi‐modal datasets.

#### 4.4.1 Comparison With SOTA Models

Based on quantitative experimental results, our proposed MFR‐UNet consistently achieved optimal or near‐optimal performance across all metrics on all four datasets, comprehensively outperforming all compared SOTA methods. This sustained competitive advantage is not coincidental but rather the inevitable outcome of its purposeful modular design synergistically addressing diverse clinical challenges.

As shown in Table 1, In the CVC‐ClinicDB dataset, polyp segmentation is challenging because of their diverse morphologies and sometimes indistinct boundaries with surrounding mucosa. MFR‐UNet achieved a leading Dice score here, primarily due to the synergistic effects of the LRFA and WCF modules. The LRFA module, through its parallel large receptive field convolutions and self‐attention mechanism, captures the complete contour and contextual information of the entire polyp. This avoids the limitation of traditional CNNs, whose restricted receptive fields result in incomplete segmentation due to a narrowed view. Simultaneously, the WCF module plays a crucial role when sampling and fusing features from the encoder at the decoder. Rather than simply concatenating features, it dynamically generates channel weights for the decoder's semantic features using the encoder's high‐resolution features. This enables the model to intelligently determine which information to prioritise in boundary regions, achieving precise delineation of polyp edges.

**TABLE 1:** Quantitative comparison with SOTA methods on the CVC‐ClinicDB and ISIC 2017 datasets.

Model	CVC‐ClinicDB				ISIC 2017			
	Dice (%)	IoU (%)	Precision (%)	Sensitivity (%)	Dice (%)	IoU (%)	Precision (%)	Sensitivity (%)
U‐Net [12]	89.72 ± 0.30	82.71 ± 0.65	90.51 ± 0.50	89.88 ± 0.21	88.15 ± 0.21	81.03 ± 0.26	90.95 ± 0.68	88.77 ± 0.92
U‐Net++ [14]	90.03 ± 0.28	83.62 ± 0.30	91.41 ± 0.29	90.95 ± 0.45	88.71 ± 0.13	81.80 ± 0.17	91.66 ± 0.41	89.01 ± 0.25
AttUnet [16]	89.79 ± 0.20	83.31 ± 0.25	91.48 ± 0.48	89.31 ± 0.50	88.59 ± 0.22	81.58 ± 0.23	90.46 ± 0.25	89.99 ± 0.30
UNeXt [29]	84.85 ± 0.42	76.50 ± 0.20	87.81 ± 0.45	84.40 ± 0.40	88.66 ± 0.20	81.78 ± 0.22	91.58 ± 0.32	89.48 ± 0.29
DualA‐Net [30]	90.21 ± 0.31	84.00 ± 0.42	92.48 ± 0.55	90.18 ± 0.22	88.65 ± 0.12	82.03 ± 0.18	92.98 ± 1.60	88.28 ± 1.55
DPMNet [31]	90.89 ± 0.22	84.75 ± 0.29	92.55 ± 0.15	90.05 ± 0.11	89.01 ± 0.15	82.35 ± 0.16	92.35 ± 0.09	89.45 ± 0.15
TPFIANet [27]	90.95 ± 0.15	84.82 ± 0.22	92.58 ± 0.11	90.11 ± 0.08	89.05 ± 0.12	82.41 ± 0.15	92.41 ± 0.05	89.55 ± 0.13
**MFR‐UNet**	**91.25** ± **0.18**	**85.05** ± **0.25**	**92.75** ± **0.08**	**91.30** ± **0.04**	**89.25** ± **0.17**	**82.60** ± **0.18**	**92.55** ± **0.02**	**89.70** ± **0.11**

*Note:* Bold indicates the best result.

On the more challenging ISIC 2017 dermatoscopy dataset, lesions like melanoma exhibit highly irregular shapes, variable colour textures, and often feature feathered blurred boundaries with healthy skin. MFR‐UNet achieved top performance on this dataset, fully demonstrating the value of the WtConv module. Traditional CNNs inevitably lose the high‐frequency information defining these irregular boundaries during successive downsampling. WtConv, however, decomposes feature maps into the frequency domain, enabling the separation and independent processing of high‐frequency components representing edges and texture. This allows the network to retain and learn these critical diagnostic details even in its deeper layers. Consequently, when reconstructing the segmentation map, the model restores boundaries that are finer and more closely aligned with the actual lesion contours than other models.

As shown in Table 2, the primary challenge in the DDTI thyroid ultrasound dataset stems from the images' inherent low contrast and intense speckle noise, which severely disrupts nodule boundary identification. MFR‐UNet also demonstrates the strongest robustness in such tasks. This success further highlights the advantages of the WtConv module, which effectively separates low‐frequency signals representing nodule structure from high‐frequency signals representing speckle noise. Furthermore, under low signal‐to‐noise conditions, the long‐range dependency modelling capability of the LRFA module becomes particularly crucial. It enables the model to integrate scattered, faint evidence across the image, forming a global judgement on nodule location and morphology rather than being misled by locally intense noise.

**TABLE 2:** Quantitative comparison with SOTA methods on the DDTI and MICCAI tooth datasets.

Model	DDTI				MICCAITooth			
	Dice (%)	IoU (%)	Precision (%)	Sensitivity (%)	Dice (%)	IoU (%)	Precision (%)	Sensitivity (%)
U‐Net [12]	75.12 ± 0.45	62.88 ± 0.51	82.01 ± 0.42	75.03 ± 0.55	92.41 ± 0.40	86.09 ± 0.53	92.68 ± 0.41	92.55 ± 0.70
U‐Net++ [14]	75.61 ± 0.41	63.55 ± 0.48	80.82 ± 0.39	75.91 ± 0.51	92.63 ± 0.15	86.41 ± 0.37	92.70 ± 0.26	92.39 ± 0.40
AttUnet [16]	74.41 ± 0.35	62.35 ± 0.39	81.12 ± 0.31	75.72 ± 0.44	92.31 ± 0.10	85.92 ± 0.15	92.20 ± 0.49	92.85 ± 0.35
UNeXt [29]	75.45 ± 0.39	63.15 ± 0.43	81.80 ± 0.36	76.71 ± 0.49	91.58 ± 0.03	84.66 ± 0.05	91.49 ± 0.15	92.10 ± 0.13
DualA‐Net [30]	76.71 ± 0.39	64.70 ± 0.42	82.95 ± 0.35	79.92 ± 0.46	91.93 ± 0.31	85.28 ± 0.40	92.57 ± 1.10	91.73 ± 0.20
DPMNet [31]	77.15 ± 0.37	64.81 ± 0.40	83.01 ± 0.33	80.65 ± 0.45	92.45 ± 0.08	86.45 ± 0.11	92.41 ± 0.25	92.85 ± 0.25
TPFIANet [27]	77.21 ± 0.35	64.85 ± 0.38	83.05 ± 0.31	80.69 ± 0.43	92.51 ± 0.05	86.51 ± 0.08	92.48 ± 0.20	92.91 ± 0.20
**MFR‐UNet**	**77.45** ± **0.32**	**65.10** ± **0.36**	**83.25** ± **0.30**	**80.85** ± **0.41**	**92.70** ± **0.01**	**86.75** ± **0.04**	**92.65** ± **0.23**	**93.05** ± **0.22**

*Note:* Bold indicates the best result.

Finally, on the MICCAITooth dental x‐ray dataset which demands exceptionally high segmentation accuracy MFR‐UNet still achieved the best results. Teeth, as high‐density tissues, feature compact structures and subtle boundaries where even minor deviations lead to incorrect segmentation. The success of MFR‐UNet stems from the synergistic interaction of all its components. LRFA provides holistic layout information of the dental arch; WtConv enhances perception of high‐frequency details such as interdental spaces and enamel edges; whereas the WCF module ensures that this multidimensional information, extracted from global, frequency‐domain and local perspectives, is fused losslessly and efficiently during the decoder's layer‐by‐layer resolution recovery process, ultimately achieving pixel‐level precision localisation.

#### 4.4.2 Ablation Studies

To validate the individual contributions of our three core modules (LRFA, WtConv and WCF) and their synergistic effects, we conducted a series of exhaustive ablation experiments on the CVC‐ClinicDB dataset using U‐Net as the baseline.

As shown in Table 3, the results demonstrate that when U‐Net serves as the baseline, adding any single module we designed (LRFA, WtConv, or WCF) to the baseline model yields performance improvements to varying degrees. Among these, the LRFA module delivers the most significant gains, strongly confirming the critical importance of introducing long‐range dependencies and global context modelling into the deep encoder layers for enhancing segmentation performance. The inclusion of WtConv and WCF modules also yielded considerable performance gains, validating their effectiveness in frequency domain analysis and feature fusion.

**TABLE 3:** Module ablation study results of MFR‐UNet on the CVC‐ClinicDB dataset.

Model variant	Dice	IoU	Precision	Sensitivity
Baseline (U‐Net)	89.72	82.71	90.51	89.88
Baseline + WtConv	90.45	83.65	91.15	90.65
Baseline + WCF	90.21	83.28	90.88	90.33
Baseline + LRFA	90.95	84.31	91.55	90.21
MFR‐UNet (w/o LRFA)	90.33	83.95	91.89	90.55
MFR‐UNet (w/o WtConv)	90.45	84.15	92.01	90.78
MFR‐UNet (w/o WCF)	90.68	84.45	92.25	90.01
**MFR‐UNet**	**91.15**	**85.05**	**92.75**	**91.30**

*Note:* Bold indicates the best result.

Notably, the combined effect of the modules is evident. Removing any single module from the complete MFR‐UNet results in a noticeable decline in performance. The greatest degradation occurs when LRFA is removed, further highlighting its central role within the architecture. Similarly, removing either WtConv or WCF also leads to performance loss, demonstrating that frequency domain analysis and adaptive feature fusion are indispensable for achieving high‐precision segmentation, they are not redundant design elements.

Ultimately, the complete MFR‐UNet model‐integrating all three modules working synergistically‐achieved the best performance, with a Dice score significantly higher than all baselines and partial combination models. This result fully demonstrates the synergistic advantages and rationality of our proposed method: the three modules are not a simple stacking of functions but rather a complementary and indispensable organic whole.

#### 4.4.3 Complexity Analysis

When evaluating segmentation model performance, computational complexity and inference speed are equally critical in clinical deployment alongside segmentation accuracy. As shown in Table 4, we comprehensively assessed the model parameters (Params), floating‐point operations (FLOPs) and single‐image inference time for MFR‐UNet and other SOTA methods.

**TABLE 4:** Complexity analysis of MFR‐UNet and SOTA methods.

Network	Params (M)	FLOPs (G)	Inference time (ms)
U‐Net (2015) [12]	7.85	56.40	**1.5**
UNet++(2018) [14]	9.44	128.39	3.7
Att‐Unet (2018) [16]	34.88	266.53	4.3
UNeXt (2022) [29]	**1.47**	**2.29**	1.7
DualA‐Net (2024) [30]	2.58	22.04	2.9
DPMNet (2024) [31]	28.04	31.76	1.9
TPFIANet (2025) [27]	10.49	256.07	2.7
MFR‐UNet	27.26	201.27	2.2

*Note:* Bold indicates the best result.

Our MFR‐UNet exhibits higher parameter counts and computational demands compared to lightweight models such as UNeXt or the baseline U‐Net. This primarily stems from integrating powerful feature refinement modules (LRFA, WtConv and WCF) at different network stages, which inevitably introduce additional computational overhead to achieve deep feature refinement. Nevertheless, we consider this increase in complexity a carefully designed and valuable trade‐off.

As demonstrated by the preceding experimental results, this moderate resource investment yields significant and consistent performance gains in segmentation accuracy, robustness, and generalisation capability. More importantly, MFR‐UNet demonstrates outstanding performance in practical inference efficiency. Despite its higher FLOPs, the highly parallelised architecture of our designed modules (e.g., LRFA and WtConv) fully leverages modern GPU computational power, keeping single‐image inference time within the range required for clinical real‐time or near‐real‐time auxiliary diagnosis. Thus, MFR‐UNet achieves a favourable trade‐off between model complexity and segmentation performance.

#### 4.4.4 Visualisation of Segmentation Results

To more intuitively demonstrate the segmentation performance advantages of our proposed MFR‐UNet, we conducted a visual analysis of segmentation results across typical cases in addition to quantitative metric comparisons. These visualisations not only corroborate our quantitative data but also reveal our model's specific strengths in handling complex boundaries, noise interference and fine‐grained structures.

**FIGURE 5:**
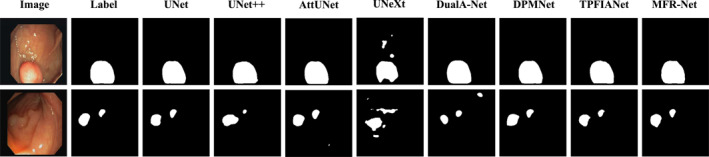
Comparison of MFR‐UNet and SOTA methods for visual segmentation on the CVC‐ClinicDB dataset.

**FIGURE 6:**
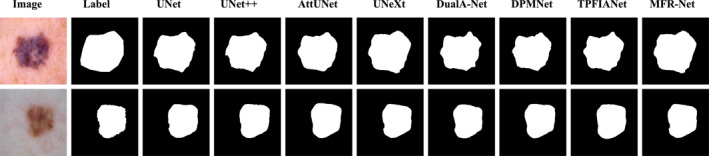
Comparison of MFR‐UNet and SOTA methods for visual segmentation on the ISIC2017 dataset.

**FIGURE 7:**
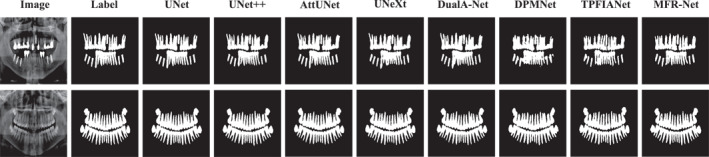
Comparison of MFR‐UNet and SOTA methods for visual segmentation on the MICCAI tooth dataset.

**FIGURE 8:**
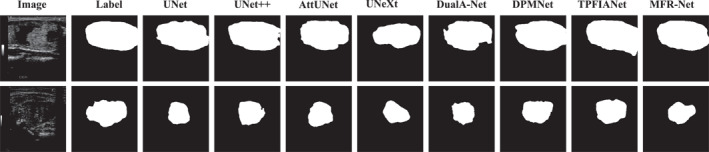
Comparison of MFR‐UNet and SOTA methods for visual segmentation on the DDTI dataset.

**FIGURE 9:**
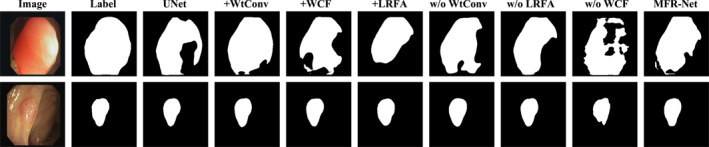
Comparison of visual segmentation for module ablation studies in MFR‐UNet.

As shown in Figures 5, 6, 7, 8, 9, comparisons with SOTA models reveal that many baseline models (e.g., U‐Net and AttUnet) struggle to fully enclose irregularly shaped polyps and skin lesions in the CVC‐ClinicDB and ISIC 2017 datasets. In contrast, the segmentation masks generated by MFR‐UNet exhibit smoother more complete contours that precisely align with lesion edges. This visually demonstrates the synergistic effect of the WtConv module in preserving high‐frequency boundary information and the LRFA module in understanding global morphology. When confronted with the intense speckle noise in DDTI images, segmentation results from other models often exhibit isolated small patches of misclassification caused by noise. MFR‐UNet, however, delivers cleaner and more robust results due to WtConv's effective separation of signal and noise.

Visualisation results from ablation experiments further reveal the indispensability of each core module. The baseline U‐Net model produces relatively coarse segmentation results. When the LRFA module is removed, the model sometimes loses grasp of the target's overall shape, leading to structurally incomplete segmentation results. When the WtConv module is removed, segmentation boundaries become noticeably blurred, failing to handle fine texture details. Removing the WCF module resulted in incomplete segmentation regions and weaker boundary coherence, indicating ineffective cross‐level feature alignment and fusion. Ultimately, the complete MFR‐UNet model delivered the closest visual results to ground truth, exhibiting optimal performance in structural integrity, boundary definition and internal consistency.

## 5 Conclusions

In this paper, we propose a novel medical image segmentation network—MFR‐UNet—aimed at addressing the core limitations of existing U‐Net architectures in global context capture, multi‐frequency domain feature processing, and cross‐level information fusion. By innovatively integrating LRFA, WtConv and WCF modules, our model performs deep refinement of feature representations across multiple dimensions.

Extensive experimental results robustly demonstrate the effectiveness and superiority of MFR‐UNet. Across four public datasets spanning diverse imaging modalities (endoscopy, dermatoscopy, ultrasound and x‐ray), MFR‐UNet consistently outperforms multiple state‐of‐the‐art methods on key performance metrics. This success stems from its modular, synergistic design: LRFA effectively captures long‐range dependencies, WtConv precisely preserves high‐frequency boundary details and WCF enables intelligent cross‐level feature fusion.

Despite MFR‐UNet's encouraging results, we recognise room for optimisation in model complexity. Future work will focus on two primary directions: First, we will explore model lightweighting techniques such as knowledge distillation and network pruning to reduce computational costs while maintaining high performance, making it more deployable in resource‐constrained clinical settings. Second, we will strive to extend MFR‐UNet's 2D framework to 3D for handling volumetric data such as MRI and CT scans, which holds greater clinical significance for tumour volume measurement and surgical planning.

## Author Contributions

**Shaoqiang Wang:** writing – original draft, validation, software, methodology, data curation. **Guiling Shi:** software, formal analysis. **Shuo Sun:** data curation. **Yuchen Wang:** methodology, formal analysis. **Yulin Zhang:** validation, supervision. **Weixian Li:** formal analysis. **Yawu Zhao:** formal analysis. **Xiaochun Cheng:** validation, supervision.

## Funding

This work was funded by Hebei Province Education Science “14th Five‐Year Plan” (Grant 2303065), UKRI (Grant EP/W020408/1), and Doctoral Training Centre at Swansea University (Grant RS718).

## Conflicts of Interest

The authors declare no conflicts of interest.

## Data Availability

All datasets used in this study are publicly accessible.
